# Esculetin and Fucoidan Attenuate Autophagy and Apoptosis Induced by Zinc Oxide Nanoparticles through Modulating Reactive Astrocyte and Proinflammatory Cytokines in the Rat Brain

**DOI:** 10.3390/toxics10040194

**Published:** 2022-04-16

**Authors:** Woo-Ju Song, Jeongtae Kim, Taekyun Shin, Myeong-Seon Jeong, Kil-Nam Kim, Jang-Hyuk Yun, Myung-Bok Wie

**Affiliations:** 1 Department of Veterinary Toxicology, College of Veterinary Medicine and Institute of Veterinary Science, Kangwon National University, Chuncheon 24341, Korea; songwooju@ncc.re.kr; 2 Center for Breast Cancer, National Cancer Center, Goyang 10408, Korea; 3 Department of Anatomy, Kosin University College of Medicine, Busan 49267, Korea; island7805@hanmail.net; 4 College of Veterinary Medicine and Veterinary Medical Research Institute, Jeju National University, Jeju 63243, Korea; shint@jejunu.ac.kr; 5 Chuncheon Center, Korea Basic Science Institute, Chuncheon 24341, Korea; jms0727@kbsi.re.kr (M.-S.J.); knkim@kbsi.re.kr (K.-N.K.); 6 Department of Biochemistry, College of Natural Sciences, Kangwon National University, Chuncheon 24341, Korea; 7 Department of Veterinary Pharmacology, College of Veterinary Medicine and Institute of Veterinary Science, Kangwon National University, Chuncheon 24341, Korea; yunjh@kangwon.ac.kr

**Keywords:** lipoxygenase, tumor necrosis factor-α, beclin-1, glial fibrillary acidic protein, hippocampus

## Abstract

We examined the protective effects of esculetin and fucoidan against the neurotoxicity of ZnO NPs in rats. Ninety rats were divided into nine groups and pre-treated with esculetin or fucoidan 1 h before ZnO NP administration on a daily basis for 2 weeks. Serum and brain homogenates were examined by enzyme-linked immunosorbent assay (ELISA), and neurons, microglia, and astrocytes in the hippocampal region were examined with immunohistochemical analysis. The serum levels of interleukin-1-beta (IL-1β), 3-nitrotyrosine (3-NT), superoxide dismutase (SOD), and 8-hydroxy-2′-deoxyguanosine (8-OHdG) were altered in the ZnO NP treatment groups. Brain IL-1β and TNF-α levels were elevated after ZnO NP administration, and these effects were inhibited by esculetin and fucoidan. SOD, 8-OHdG, and acetylcholinesterase (AChE) levels in the brain were decreased after ZnO NP administration. The brain levels of beclin-1 and caspase-3 were elevated after ZnO NP treatment, and these effects were significantly ameliorated by esculetin and fucoidan. The number of reactive astrocytes measured by counting glial fibrillary acidic protein (GFAP)-positive cells, but not microglia, increased following ZnO NP treatment, and esculetin and fucoidan ameliorated the changes. Esculetin and fucoidan may be beneficial for preventing ZnO NP-mediated autophagy and apoptosis by the modulation of reactive astrocyte and proinflammatory cytokines in the rat brain.

## 1. Introduction

Nanotechnology has been applied extensively in the fields of electronics, construction, consumer products, medical devices, medicines, foodstuffs, and environmental remediation, although these engineered nanomaterials have potential toxicity [1,2,3]. Nanoparticles (NP) are generally known to exhibit unique and highly reactive physicochemical properties. Accordingly, the possibility of NP invasion into brain through the blood–brain barrier (BBB) [4,5] and nasal olfactory pathway [6] remains a concern and can cause neurodegenerative disorders, such as Parkinson and Alzheimer’s disease [5,7,8]. Conversely, various NPs have also attracted attention as useful therapeutics, as well as for antibacterial and anticancer purposes [9,10]. Therefore, it is essential to establish guidelines for in vivo neurotoxic concentrations of NPs. Zinc oxide NPs (ZnO NPs) have been utilized as food additives, as well as in cosmetics, in medicinal drugs in the pharmaceutical industry, and in biotechnology products [11,12,13]. Zinc is an essential trace metal that plays roles in several pathophysiological conditions in the central nervous system, including seizure, ischemic stroke, and AD [14,15,16,17,18]. In particular, zinc is known to accumulate at high levels in synaptic vesicles and mossy fibers in the hippocampus, a brain region with important roles in learning and memory [19,20]. Recently, it has been reported that ZnO NPs induce proinflammatory cytokine-mediated neuroinflammation and apoptosis, including oxidative damage, after their oral administration in rats [21]. The administration of ZnO NPs through various routes, including intraperitoneal injection, instillation on the tongue, and oral gavage, has been shown to induce an impairment of the memory function in rodents [22,23,24,25]. The neurotoxic mechanism of action of ZnO NPs by oral administration is known to involve the production of reactive oxygen species (ROS) or reactive nitrogen species, a reduction in antioxidant enzymes (glutathione [GSH], superoxide dismutase [SOD], catalase [CAT]), an elevation of proinflammatory cytokines (interleukin-1-beta [IL-1β], tumor necrosis factor-alpha [TNF-α]), and acceleration of apoptosis (caspase-3, Fas, DNA fragmentation) in brain tissues, resulting in learning and memory deficits [21]. Moreover, the intraperitoneal injection of ZnO NPs was shown to induce gliosis and necrosis, the cerebral accumulation of zinc, and elevated P53 and cyclooxygenase-2 (COX-2) expression in some brain areas (cerebellar cortex, cerebral cortex, and hippocampus) [22,26]. Therefore, anti-inflammatory and antioxidant compounds are used to relieve ZnO NP-mediated neuropathological conditions. Previously, we reported that ZnO NPs induce apoptosis and necrosis in human neuroblastoma SH-SY5Y cells mediated by lipoxygenase (LOX), but not cyclooxygenase-2 (COX-2) [27]. Here, esculetin was shown to have neuroprotective effects against ZnO NP-induced neurotoxicity. Esculetin (6,7-dihydroxycoumarin and cichorigenin, CAS 305-01-1) is known as a phenolic compound and coumarin derivative found in various natural plants. Its pharmacological properties are antioxidant [28], anti-inflammatory [29], antiapoptotic [30], and neuroprotective [31]. However, both inhibitors of LOX and COX-2 attenuated ZnO NP-mediated toxicity in primary astrocyte cultures and human bone marrow-derived mesenchymal stem cells [32,33]. These results suggested that astrocytes show greater vulnerability to the toxicity of ZnO NPs than neuronal cells. Arachidonate metabolism-associated enzymes, such as LOX and COX-2, may respond differently to ZnO NP-mediated toxicity according to the cell type. Therefore, we examined whether the selective LOX inhibitor, esculetin, would show neuropharmacological availability in vivo, and protect astrocytes in the hippocampal region against ZnO NP-mediated neurotoxicity. Fucoidan (sulfated fucans) is a natural product extracted from edible brown seaweeds containing sulfated polysaccharides and alginate, which are chiefly composed of fucose and sulfate residues [34]. Fucoidan is known to exhibit various biological and pharmacological activities, such as anticancer [35], anti-inflammatory [36], neuroprotective activities [37]. In addition, we also examined whether fucoidan exhibits neuroprotective activity against ZnO NP-induced neurotoxicity.

## 2. Material and Methods

### 2.1. Animals and Test Chemicals

Ninety female Sprague–Dawley rats, aged 6 weeks, and weighing 170 ± 10 g were obtained from Orient Bio Inc. (Seongnam-si, Gyeonggi-do, Korea). Animals were kept under standard conditions (12 h light/dark cycle, temperature 22 ± 2 °C, and relative humidity 50 ± 10%) and provided with standard food pellets and tap water ad libitum. All animal experimental procedures were approved by the Institutional Animal Care and Use Committee (IACUC) (Approval No. KW-201029-1). We selected the female rats due to gender equity and their nature of being less territorial. The 90 rats were divided into the following nine groups: untreated control; 10 mg/kg of ZnO NPs; 100 mg/kg of ZnO NPs; 100 mg/kg of ZnO NPs + 5 mg/kg of esculetin; 100 mg/kg of ZnO NPs + 25 mg/kg of esculetin; 25 mg/kg of esculetin only; 100 mg/kg of ZnO NPs + 10 mg/kg of fucoidan; 100 mg/kg of ZnO NPs + 30 mg/kg of fucoidan; 30 mg/kg of fucoidan only. Rats were orally pre-treated with esculetin or fucoidan 1 h before ZnO NP administration once daily for 2 weeks, and the body weight of the rats was checked every 2 days.

ZnO NP was dispersed in a saline solution and the suspension was sonicated for 30 min before administration to prevent agglomeration. After 2 weeks, rats were sacrificed under anesthesia with tribromoethanol, and blood samples were collected from the abdominal aorta for analysis. Serum from blood was separated by standing at room temperature for 30 min and centrifugation at 3000× *g* for 10 min. The collected brains were weighed and stored at −80 °C until enzyme-linked immunosorbent assay (ELISA) and inductively coupled plasma-optical emission spectrometry (ICP-OES). For an immunohistochemical analysis, rats were anesthetized with tribromoethanol (300 mg/kg, i.p) and perfused transcardially with 0.1 M phosphate-buffered saline (PBS, pH 7.4) followed by 4% paraformaldehyde solution. The brains were removed and postfixed in the same fixatives overnight. Fucoidan was purchased from Sigma Chemical Co. (St. Louis, MO, USA) and derived from the marine algae, *Fucus vesiculosus*. Esculetin was purchased from Enzo Life Sciences (Farmingdale, NY, USA). For administration into rats, fucoidan and esculetin were dissolved in a saline solution. The solutions were vortexed vigorously before every treatment.

### 2.2. Production and Characterization of ZnO NPs

ZnO NPs (Lot No.: D28X017, Cat. No. 44898, ZnO NPs, ZnO NanoGard^®^) were purchased from Alfa Aesar Co. (Ward Hill, Haverhill, MA, USA); the average particle size of the ZnO NPs in the powder was 67 nm (40–100 nm), and the range of the specific surface area (based on the Brunauer-Emmett-Teller [BET] theory) was 16 m^2^/g (10–25 m^2^/g).

### 2.3. Assessment of Zinc Content

The rats were sacrificed as described above, the brains were weighed, and immediately frozen and stored at −80 °C. Brain homogenates were digested in HNO_3_ at 160 °C for 3 h. All measurements were performed by ICP-OES (Agilent 5900; Agilent Technologies, Santa Clara, CA, USA) with nebulizer, plasma, and aux flow rates of 0.7, 12, and 1 L/mL, respectively. The operation conditions were adjusted for optimal determination. Calibration curves were prepared separately by running suitable concentrations of standard solutions. Control blanks were also prepared and analyzed in the same manner. The average values of six replicates were obtained and the zinc concentration was calculated in μg/g of dry mass.

### 2.4. ELISA Assay

The levels of interleukin-1-beta (IL-1β), tumor necrosis factor- α (TNF-α), catalase (CAT), superoxide dismutase (SOD), 8-hydroxy-2′-deoxyguanosine (8-OHdG), 3-nitrotyrosine (3-NT), acetylcholinesterase (AChE), beclin-1, and caspase-3 were measured using rat ELISA kits (Cusabio, Wuhan, China) in accordance with the manufacturer’s instructions. Briefly, the serum levels of IL-1β, TNF-α, CAT, SOD, 8-OHdG, and 3-NT were assessed by ELISA in samples of 100 μL of serum collected from blood. To assess the levels of IL-1β, TNF-α, CAT, SOD, 8-OHdG, AChE, beclin-1, and caspase-3 in brain homogenates, brain tissue was treated with Pierce™ RIPA Buffer (Thermo Fisher Scientific, Waltham, MA, USA) and Halt™ Protease Inhibitor Cocktail (Thermo Fisher Scientific), and homogenized on ice with 30-s ON/3-s OFF cycles. The cycle was repeated three times. The homogenates were centrifuged at 12,000 rpm for 20 min at 4 °C, and 100 μL of supernatant was transferred to a fresh tube. Then, 100 μL of serum (for IL-1β, TNF-α, CAT, SOD, 8-OHdG, and 3-NT) or brain homogenate (for IL-1β, TNF-α, CAT, SOD, 8-OHdG, AChE, beclin-1, and caspase-3) and standards were added to 96-well microplates coated with specific antibodies and incubated at 37 °C in a 5% CO_2_ incubator for 2 h. After 2 h, the supernatant was removed, 100 μL of 1× biotin antibody was added to each well, and the plates were incubated at 37 °C in a 5% CO_2_ incubator for 1 h. Each well was then washed three times with 1× wash buffer, 100 μL of 1× horseradish peroxidase, after which avidin was added, and the plates were incubated at 37 °C in a 5% CO_2_ incubator for 1 h. Each well was washed five times, 90 μL of 3,3′,5,5′-tetramethylbenzidine was added as peroxidase substrate, and the plates were incubated at 37 °C in a 5% CO_2_ incubator for 25 min in the dark. Following the addition of 50 μL of stop solution, the levels of IL-β, TNF-α, CAT, SOD, 8-OHdG, 3-NT, AChE, beclin-1, and caspase-3 were determined by measuring the absorbance at 450 nm (A_450_) using a VersaMax microplate reader (Molecular Devices, Downingtown, PA, USA).

### 2.5. Protein Assay

To measure the levels of protein in brain tissue, a bicinchoninic acid Protein Assay Kit (Takara Bio, Inc., Nojihigashi, Japan) was used according to the manufacturer’s instructions. The supernatant was obtained from homogenized and centrifuged brain tissue, and 100 μL of supernatant, standard, and the working solution were added to a 96-well microplate and incubated at 60 °C for 1 h. After 1 h, the amount of protein was determined by measuring the absorbance at 562 nm (A_562_) using a VersaMax microplate reader (Molecular Devices).

### 2.6. Transmission Electron Microscopy

Prior to transmission electron microscopy (TEM), the rat brain tissue was washed with 0.1 M PBS and fixed with a mixture of 2% paraformaldehyde and 2% glutaraldehyde for 2 h at 4 °C. The tissue was post-fixed in 1% osmium tetroxide in the same buffer, and dehydrated with ethanol and propylene oxide. Subsequently, the samples were embedded in Epon-812 resin and ultrathin sections were cut using an ultramicrotome (Leica Microsystems, Wetzlar, Germany). Finally, sections were stained with uranyl acetate and lead citrate and visualized by TEM (JEM-2100F; JEOL, Tokyo, Japan).

### 2.7. Tissue Preparation and Histological Examination

Brains were fixed with 4% paraformaldehyde in phosphate buffer (0.1 M, pH 7.4). Samples were embedded in paraffin, and sections 5 µm thick were cut using a rotary microtome (Leica RM 2135; Leica Microsystems). Paraffin sections were deparaffinized in xylene and dehydrated by passing through an ethanol series, followed by immunohistochemical labeling and staining with hematoxylin and eosin (H&E).

### 2.8. Immunohistochemistry

Immunohistochemistry was performed using a Vectastain Elite ABC Kit (Vector Laboratories, Burlingame, CA, USA), as described previously [38]. Briefly, deparaffinized sections were treated with citrate buffer (0.01 M, pH 6.0) in a microwave for 3 min for antigen retrieval, and then treated with 0.3% hydrogen peroxide in methyl alcohol for 20 min to block endogenous peroxidase activity. Subsequently, sections were incubated with the appropriate blocking serum (10% [*v/v*] goat, rabbit, or horse serum in PBS; Vectastain Elite ABC Kit; Vector Laboratories). The samples were incubated with primary antibodies, including rabbit anti-ionized calcium binding adaptor molecule-1 (Iba1) (019-19749; Wako, Tokyo, Japan) and glial fibrillary acidic protein (GFAP) (G3893; Sigma-Aldrich, St. Louis, MO, USA) for 1 h at room temperature. After three washes with PBS, sections were incubated with biotinylated rabbit IgG antibody and then with ABC peroxidase, according to the manufacturer’s instructions. The peroxidase reaction was developed using a 3-3′-diaminobenzidine substrate kit (Vector Laboratories), followed by counterstaining with hematoxylin before mounting. Immunostained sections (three different animals per group) were photographed under a 4× magnification objective lens using a ProgRes C5 digital camera (Olympus DP72; Olympus Corp., Tokyo, Japan) attached to a light microscope (Olympus BX53/U-LH 100HG; Olympus Corp.). The equipment for visualization used in the histological studies was ZEISS Axio scan.Z1 slide microscope (Serial No. 4646000307; Oberkochen, Germany) and the ZEN 3.4 program. Immunostaining was semi-quantified based on the brown-colored areas in the photographs using ImageJ software (NIH, Bethesda, MD, USA). For semi-quantitative analysis, Iba1-postivie cells were counted in three different rats in each group, and the GFAP-positive area was measured as a percentage of the stained area [(positive area/total area) × 100 (%)]. All values are presented as the mean ± standard error of the mean (SEM) and were subjected to one-way analysis of variance (ANOVA) followed by Student–Newman–Keuls post hoc test for multiple comparisons, with *p* < 0.05 indicating statistical significance.

### 2.9. Statistical Analysis

All statistical analyses were performed using the SAS 9.4 (SAS Institute, Cary, NC, USA). Statistical analyses consisted of one-way ANOVA and Tukey’s multiple comparison test. All experimental data are expressed as the mean ± SEM. All experiments were performed at least three times with similar results. In all analyses, *p* < 0.05 was taken to indicate statistical significance.

## 3. Results and Discussion

### 3.1. Changes in the Body and Organ Weights and Brain Zinc Concentration by ZnO NP Administration after Pre-Treatment of Esculetin or Fucoidan

**Figure 1:**
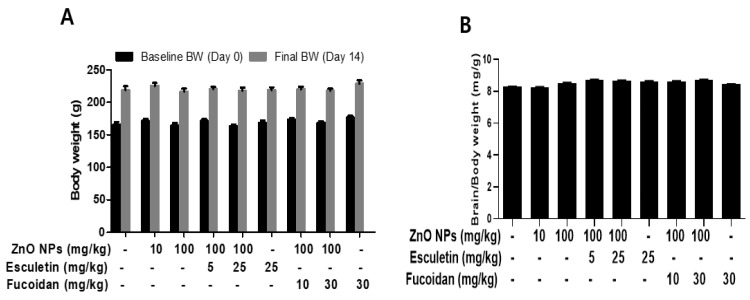
Effects of esculetin and fucoidan on body (**A**) and brain (**B**) weights in ZnO NP-treated rats. Data represent the mean ± SEM (*n* = 10).

The body weights of rats were measured every 2 days until the end of the experimental period (2 weeks) in all groups. There were no significant changes in the body weight or relative brain weight between groups during the 2-week study period (Figure 1A,B).

**Figure 2:**
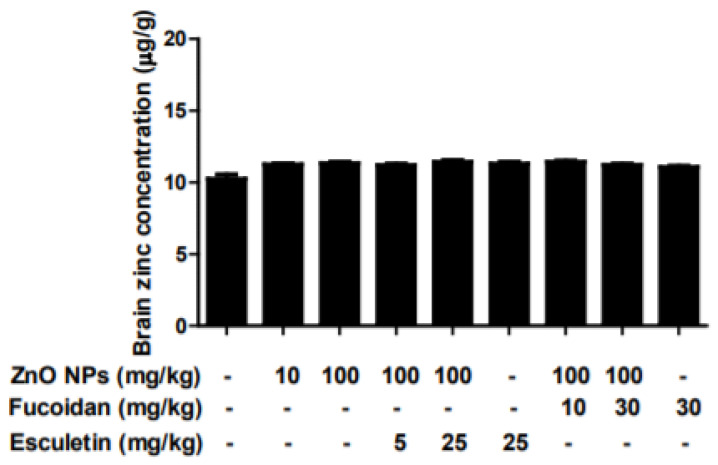
Effects of esculetin and fucoidan on zinc concentration (μg/g) in rat brain homogenates from ZnO NP-treated rats. Metallic zinc concentration was measured by ICP-OES. Data represent the mean ± SEM (*n* = 6).

The basal average zinc concentration in rats was 10.24 ± 0.29 μg/g brain tissue (Figure 2). Brain concentration of zinc was slightly increased in the groups treated with 10 and 100 mg/kg ZnO NPs for 2 weeks. However, there were no significant changes in any groups. In this study, there were no alterations in the body and brain weights or brain zinc concentration after the administration of ZnO NPs at 10 mg/kg/day or 100 mg/kg/day for 2 weeks. The intravenous (IV) injection of ZnO NPs (25 mg/kg/day) for 2 weeks was reported to significantly increase zinc concentrations in serum and brain, with no effect on body weight gain or relative brain weight and no cognitive abnormalities in rats [39]. Liang et al. [40] reported that exposure to ZnO NPs at 50 mg/kg/day by oral gavage did not alter zinc concentrations in the brain or blood. These results were similar to our observations. In contrast, tongue and intratracheal instillation of ZnO NPs were associated with significant changes in various brain regions, such as the brainstem, hippocampus, and cerebral cortex [41,42]. These results suggest that zinc accumulation in the brain may be dependent on the route of ZnO NPs administration, followed by the triggering of neurotoxic injury. That is, oral exposure to ZnO NPs was safer than non-oral exposure, such as intraperitoneal (IP), IV, tongue, or intratracheal instillation routes [26,39,40,41].

### 3.2. Changes in Serum Proinflammatory Cytokines, IL-1β and TNF-α, by ZnO NP Administration after Pre-Treatment of Esculetin or Fucoidan

**Figure 3:**
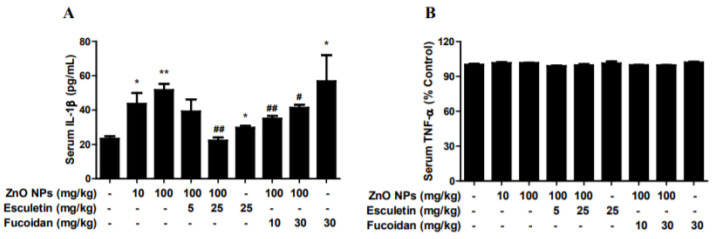
Effects of treatment with esculetin or fucoidan on serum proinflammatory cytokines, IL-1β and TNF-α, in ZnO NP-exposed rats. ZnO NP administration dose-dependently increased serum IL-1β (**A**), but not TNF-α (**B**). Esculetin and fucoidan significantly inhibited the elevation of IL-1β induced by ZnO NPs. Data represent the mean ± SEM. * *p* < 0.05, ** *p* < 0.01 compared to control. # *p* < 0.05, ## *p* < 0.01 compared to ZnO NPs (100 mg/kg) group (*n* = 6).

The oral administration of ZnO NPs at 10 and 100 mg/kg for 2 weeks was associated with a significant elevation of serum IL-1β level (Figure 3A). Esculetin (25 mg/kg) and fucoidan (10 and 30 mg/kg) significantly ameliorated these effects of ZnO NPs. Unusually marked increases in serum IL-1β were observed in the fucoidan-only treatment group. Accordingly, it would be necessary to clarify the exact etiology for certain toxicities of fucoidan itself in the near future. In contrast, ZnO NPs did not alter serum TNF-α levels at either 10 or 100 mg/kg in any of the groups (Figure 3B). Esculetin and fucoidan also did not affect these levels (Figure 3B).

### 3.3. Changes in Serum CAT, SOD, 3-NT, and OHdG after ZnO NP Administration after Pre-Treatment of Esculetin or Fucoidan

**Figure 4:**
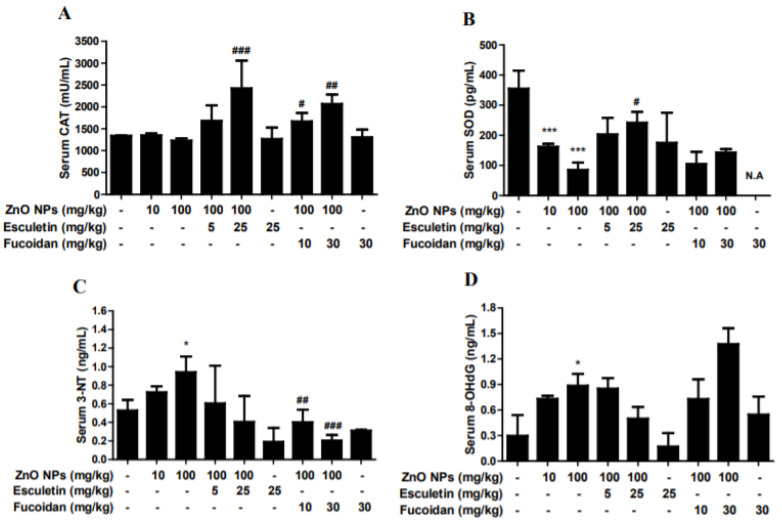
Effects of esculetin and fucoidan on serum antioxidant enzymes (**A**,**B**) and oxidative stress biomarkers (**C**,**D**) in ZnO NP-exposed rats. ZnO NP administration significantly decreased serum SOD (**B**), but not CAT (**A**). Esculetin (25 mg/kg) ameliorated the reduction of SOD level. However, esculetin and fucoidan significantly increased CAT level compared to controls. ZnO NP dose-dependently increased 3-NT (**C**) and 8-OHdG levels in serum. Esculetin ameliorated the changes in 3-NT and 8-OHdG levels, while fucoidan ameliorated only the change in 3-NT level. Data represent the mean ± SEM. * *p* < 0.05, *** *p* < 0.001 compared to control. # *p* < 0.05, ## *p* < 0.01, ### *p* < 0.001 compared to ZnO NPs (100 mg/kg) group (*n* = 6). N.A. = not assayed.

ZnO NPs (10 and 100 mg/kg) did not affect serum CAT level (Figure 4A). However, esculetin (5 and 25 mg/kg) and fucoidan (10 and 30 mg/kg) increased CAT levels in a dose-dependent manner compared to the ZnO NP (100 mg/kg) treatment group (Figure 4A). In contrast, ZnO NPs decreased SOD levels in a concentration-dependent manner compared to the control group (Figure 4B). Esculetin, but not fucoidan, reversed these effects, compared to the ZnO NP (100 mg/kg) treatment group (Figure 4B). However, serum levels of 3-NT, a marker of nitrogen-free radical species, and serum 8-OHdG, a biomarker of oxidative stress and DNA damage, were increased in a dose-dependent manner by ZnO NP administration, and the effects on both markers were statistically significant at a ZnO NP dose of 100 mg/kg (Figure 4C,D). Both esculetin and fucoidan reduced 3-NT levels compared to the ZnO NP (100 mg/kg) treatment group (Figure 4C). However, esculetin, but not fucoidan, ameliorated the effects on 8-OHdG levels compared to ZnO NP (100 mg/kg) treatment alone (Figure 4D). Furthermore, 3-NT is a well-known biomarker of peroxynitrite-mediated oxidative injury in various tissues and serum [43]. ZnO NP treatment increased serum 3-NT levels in a dose-dependent manner, while both esculetin and fucoidan reduced the 3-NT levels. Additionally, 8-OHdG, a biomarker of oxidative DNA damage and carcinogenesis, may be applicable for monitoring of serum, urine, and tissue samples in various neurodegenerative diseases [44,45]. Accordingly, it has been used for diagnostic purposes in exposure to various occupationally relevant NPs, including ZnO NPs [46,47]. In this study, 8-OHdG showed different patterns of changes between serum and brain. That is, ZnO NP administration significantly reduced 8-OHdG in the brain, but dose-dependently elevated 8-OHdG in serum. These results suggested that serum 8-OHdG may be a more reliable or sensitive marker of oxidative DNA damage than brain 8-OHdG. Esculetin, but not fucoidan, contributed to the amelioration of oxidative DNA damage after ZnO NP administration. However, both esculetin and fucoidan themselves induced additional decreases in brain 8-OHdG levels.

### 3.4. Changes in Brain Proinflammatory Cytokines and Antioxidant Enzymes by ZnO NP Administration of Pre-Treatment of Esculetin or Fucoidan

**Figure 5:**
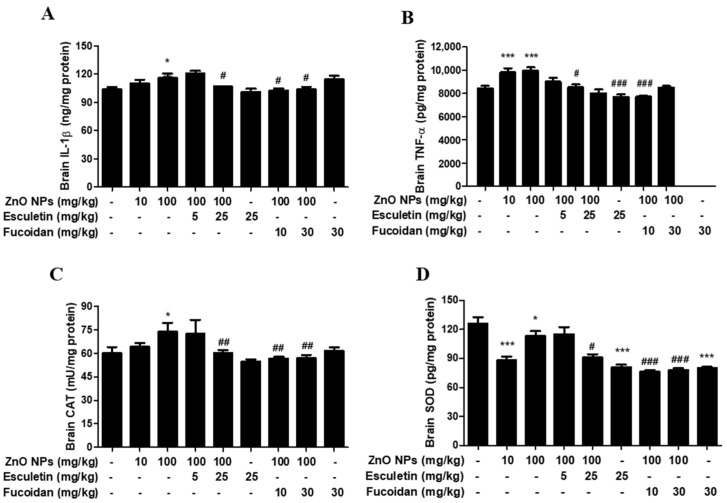
Effects of esculetin or fucoidan treatment on proinflammatory cytokines, brain IL-1β (**A**) and TNF-α (**B**), and antioxidant enzymes, CAT (**C**) and SOD (**D**), in ZnO NP-exposed rats. ZnO NP administration significantly increased brain IL-1β (**A**) and TNF-α (**B**) compared to controls. Esculetin and fucoidan significantly inhibited the elevation of brain IL-1β and TNF-α induced by ZnO NPs. Brain CAT was elevated by ZnO NP (100 mg/kg) administration, but SOD was significantly decreased by ZnO NP exposure. Esculetin and fucoidan contributed to normalization of CAT levels, but not SOD levels. Data represent the mean ± SEM. * *p* < 0.05, *** *p* < 0.001 compared to control. # *p* < 0.05, ## *p* < 0.01, ### *p* < 0.001 compared to ZnO NPs (100 mg/kg) group (*n* = 6).

The brain concentrations of proinflammatory cytokines, IL-1β and TNF-α, increased significantly following ZnO NP administration (Figure 5A,B). Esculetin (25 mg/kg) and fucoidan (10 and 30 mg/kg) significantly reduced the levels of both cytokines (Figure 4A,B). The endogenous antioxidant enzyme, CAT, was significantly elevated in the brain, in the group treated with 100 mg/kg ZnO NPs (Figure 5C). Both esculetin and fucoidan returned CAT to the control level compared to the group treated with 100 mg/kg ZnO NP alone (Figure 5C). However, the brain SOD levels were found to be significantly decreased by 10 and 100 mg/kg ZnO NP treatment, and neither esculetin nor fucoidan altered these effects (Figure 5D). Attia et al. [21] reported that oral ZnO NP treatment for 7 days increased brain proinflammatory cytokine levels (IL-1β and TNF-α) and apoptosis (caspase-3, DNA fragmentation), and decreased brain antioxidant enzyme levels (SOD, CAT). Although the exposure period to ZnO NPs was somewhat shorter in their study compared to the present study (7 days vs. 14 days, respectively), most of their data were consistent with our results, except for the CAT level. In contrast to the corresponding upregulation of IL-1β induced by ZnO NPs in the brain and serum, the levels of TNF-α were altered by ZnO NPs only in the brain and not in the serum. Ansar et al. [42] reported that serum TNF-α level was significantly elevated by the oral administration of ZnO NPs at a dose of 600 mg/kg in rats. The discrepancy between these results and those of the present study may have been due to the sixfold difference in dose of ZnO NPs. Both COX-2 and LOX are known to be involved in the arachidonate cascade in neuroinflammation-mediated neurodegenerative diseases, such as AD and Parkinson’s disease [48]. After conducting in vitro studies, we identified the greater availability of esculetin, a selective LOX inhibitor, in ZnO NP-induced neurotoxicity than COX-2 inhibitors in human dopaminergic SH-SY5Y cells [27]. In this in vivo study, esculetin also showed excellent effects against the elevation of brain IL-1β and TNF-α levels induced by ZnO NPs, and concurrently ameliorated reactive astrogliosis in the rat hippocampus. Unexpectedly, however, microglia and neuronal cells were not significantly affected by ZnO NP treatment at doses between 10 and 100 mg/kg/day despite increases in proinflammatory cytokines and apoptosis. With regard to antioxidant enzymes, the SOD levels in brain and serum decreased simultaneously with all doses of ZnO NPs examined. Esculetin, but not fucoidan, slightly ameliorated the decrease in serum SOD level compared to the 100 mg/kg/day ZnO NP treatment group, but neither of these compounds showed significant effects on this effect of ZnO NPs. Unexpectedly, the CAT levels increased in the brain after treatment with 100 mg/kg/day of ZnO NPs, but no changes were observed in serum. Esculetin and fucoidan markedly increased CAT levels in serum. However, they decreased brain CAT to a level similar to the control group. This divergence in changes in CAT levels between serum and brain may be ascribed to the differences in sensitivity, according to dose of ZnO NPs, compared with previous reports [21,42].

### 3.5. Changes in Brain 8-OHdG and AChE by ZnO NP Administration of Pre-Treatment of Esculetin or Fucoidan

**Figure 6:**
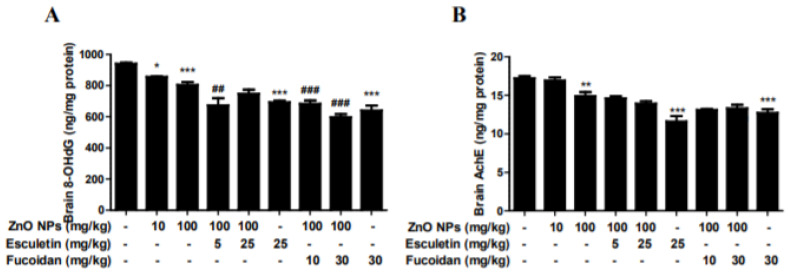
Effects of esculetin and fucoidan on ZnO NP-induced alterations in brain 8-OHdG (**A**) and AChE (**B**) levels in rats. ZnO NP administration dose-dependently decreased 8-OHdG concentration. Esculetin and fucoidan reduced 8-OHdG compared to the ZnO NP (100 mg/kg) treatment group. Brain AChE levels were significantly decreased by treatment with 100 mg/kg ZnO NPs. However, esculetin and fucoidan did not ameliorate this reduction in brain AChE level. Data represent the mean ± SEM. * *p* < 0.05, ** *p* < 0.01, *** *p* < 0.001 compared to control. ## *p* < 0.01, ### *p* < 0.001 compared to ZnO NPs (100 mg/kg) group (*n* = 6).

Interestingly, the brain contents of 8-OHdG dose-dependently decreased in accordance with ZnO NP (10 and 100 mg/kg) treatment (Figure 6A). Both esculetin and fucoidan also reduced brain 8-OHdG contents compared to the ZnO NP (100 mg/kg) treatment group (Figure 6A). The contents of brain AChE, a cognitive biomarker, significantly decreased in the 100 mg/kg ZnO NP treatment group compared to controls, and neither esculetin nor fucoidan ameliorated this effect (Figure 6B). AChE is a biomarker for diagnosis of AD. Increased AChE activity in the brain reduces the levels of ACh, resulting in behavioral impairment. Zinc homeostasis may be critical during AChE inhibitor therapy [49,50]. Conversely, ZnO NP treatment at 100 mg/kg/day significantly reduced AChE activity in the brain, suggesting that this dose of ZnO NPs may have a beneficial effect on cognitive function. Neither esculetin nor fucoidan affected this decrease in AChE activity following ZnO NP treatment.

### 3.6. Changes in Brain Autophagy and Apoptosis by ZnO NP Administration after Pre-Treatment of Esculetin or Fucoidan

**Figure 7:**
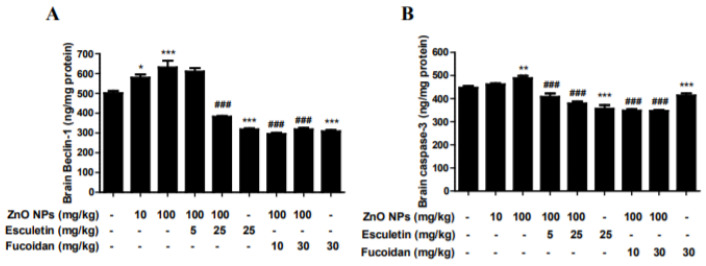
Effects of esculetin or fucoidan treatment on the brain autophagy biomarker, beclin-1, and apoptosis biomarker, caspase-3, in ZnO NP-exposed rats. ZnO NP administration dose-dependently increased brain beclin-1 (**A**) and caspase-3 (**B**) levels. Esculetin and fucoidan significantly inhibited these increases in beclin-1 and caspase-3 levels induced by ZnO NPs. Data represent the mean ± SEM. * *p* < 0.05, ** *p* < 0.01, *** *p* < 0.001 compared to control. ### *p* < 0.001 compared to ZnO NPs (100 mg/kg) group (*n* = 6).

The brain contents of the autophagy biomarker, beclin-1, significantly increased after treatment with ZnO NPs in a dose-dependent manner (Figure 7A). Both esculetin and fucoidan strongly decreased the level of this autophagy marker in the groups treated with ZnO NP (Figure 7A). Both esculetin (25 mg/kg) and fucoidan (30 mg/kg) themselves exhibited autophagy-relieving effects compared to the control group (Figure 7A). The brain contents of the apoptotic biomarker, caspase-3, were increased significantly in the group treated with ZnO NPs at 100 mg/kg (Figure 7A). Both esculetin (5 and 25 mg/kg) and fucoidan (10 and 30 mg/kg) significantly inhibited these ZnO NP-induced increases in caspase-3 level in the brain (Figure 7B). Previously, we found that ZnO NPs increased autophagic LC3-positive cells and autophagolysosomes and induced ER swelling in astrocyte cultures [32]. Autophagy is known to be involved in cellular catabolism and recycling, and plays an important role in neuronal survival [51]. Recently, the dysfunction of autophagy was shown to play a major role in the pathogenesis of neurodegenerative diseases [52]. In this study, esculetin and fucoidan potently inhibited the elevation of brain beclin-1 level, a biomarker of autophagy, induced by ZnO NPs to 40–50%. These observations suggest that autophagy may be a more sensitive indicator of early cerebral injury than apoptosis in ZnO NP-mediated neurotoxicity. Caspase-3, an apoptosis factor, was elevated in the rat brain homogenates only in the group treated with ZnO NPs at 100 mg/kg/day, and both esculetin and fucoidan ameliorated apoptosis in comparison to the groups treated with ZnO NPs only. Similar to esculetin, fucoidan is also a potential candidate agent in the regulation of ZnO NP-mediated brain injury and neurodegenerative disease [53]. In a previous study, we compared toxicity between ZnO and ZnO NPs in human bone marrow-derived mesenchymal stem cells. Here, we did not observe the differences between two materials although depolarized dead cells in the ZnO NP group were two-fold higher than in the ZnO group at a toxic concentration in the mitochondrial membrane potential results [33].

### 3.7. Changes in Brain TEM Findings by ZnO NP Treatment

**Figure 8:**
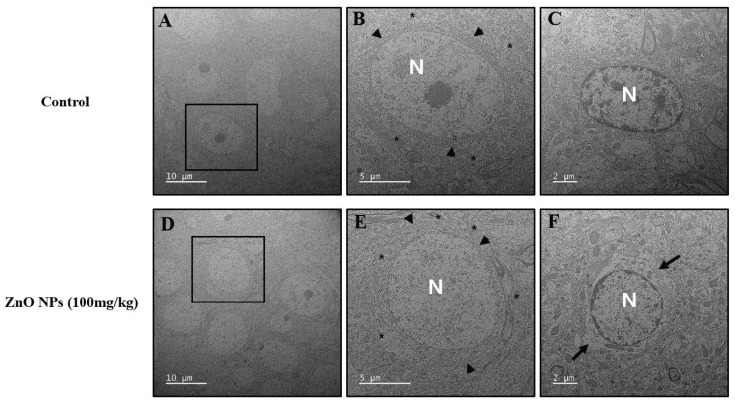
Transmission electron microscopy (TEM) images of rat hippocampus treated with vehicle as a control (**A**–**C**) or ZnO NPs (**D**–**F**). Representative hippocampal neurons (**B**,**E**) are shown in higher magnification views of the black squares in (**A**,**D**). The endoplasmic reticulum (arrowhead) and mitochondria (asterisk) were intact (**B**,**E**). Cytoplasmic swelling (arrow) (**F**) of astrocytes in the ZnO NP treatment group showed a clear distinction compared to control (**C**). N: nucleus.

The ultrastructural morphology of rat neurons in the hippocampal region did not show distinctive changes associated with oral administration of ZnO NPs for 2 weeks at a dose of 100 mg/kg (Figure 8D,E) compared to the untreated control group (Figure 8A,B). There were no differences between these two groups in the endoplasmic reticulum (ER) or mitochondria (Figure 8B,E). However, ZnO NP treatment induced astrocyte swelling (Figure 8F) compared to the control group (Figure 8C).

### 3.8. Histological Examination of the Hippocampus with H&E Staining by ZnO NPadministration after Pre-Treatment of Esculetin or Fucoidan

**Figure 9:**
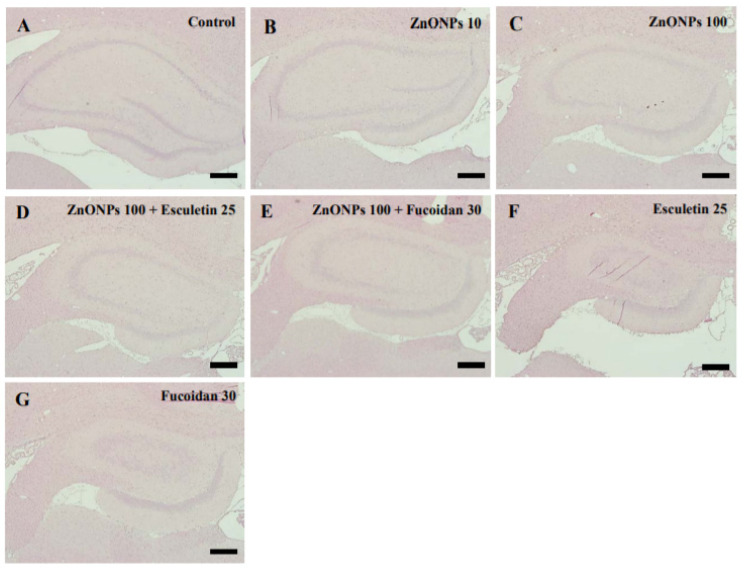
Histopathological examination by H&E staining in the hippocampus of control and ZnO NP-treated rats with or without esculetin (25 mg/kg) or fucoidan (30 mg/kg) treatment. Control (**A**), ZnO NPs (10 mg/kg) (**B**), ZnO NPs (100 mg/kg) (**C**), ZnO NPs (100 mg/kg) + esculetin (25 mg/kg) (**D**), ZnO NPs (100 mg/kg) + fucoidan (30 mg/kg) (**E**), esculetin (25 mg/kg) only (**F**), fucoidan (30 mg/kg) only (**G**). Scale bars = 200 μm.

The hippocampus, consisting of the cornu ammonis (CA) and dentate gyrus (DG), was examined in all groups (Figure 9A–G). Sections of the hippocampus from rats in the ZnO NP (10 and 100 mg/kg) treatment groups showed the same histological architecture as the untreated controls (Figure 9A–C). In addition, there were no differences in the hippocampus in the combined esculetin or fucoidan and ZnO NP treatment groups (Figure 9D,E) or the groups treated with esculetin or fucoidan alone (Figure 9F,G) compared to controls.

### 3.9. Histological Examination of the Hippocampus with Anti-NSE Antibody Staining by ZnO NP Administration after Pre-Treatment of Esculetin or Fucoidan

**Figure 10:**
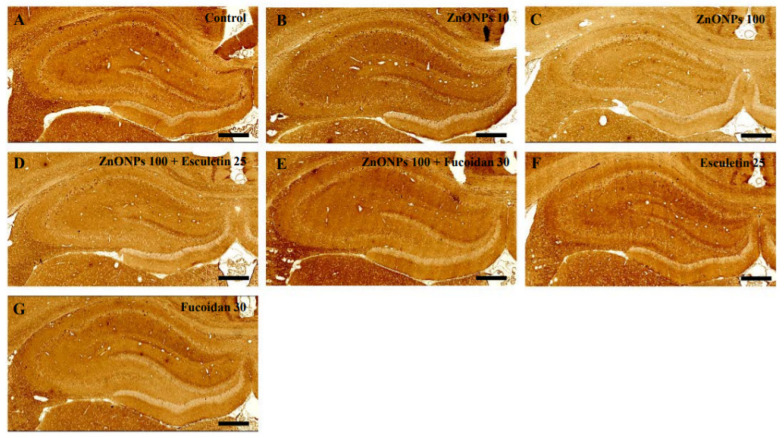
Histopathological examination by immunostaining with anti-NSE antibody in the hippocampus of control and ZnO NP-treated rats with or without esculetin (25 mg/kg) or fucoidan (30 mg/kg) treatment. Control (**A**), ZnO NPs (10 mg/kg) (**B**), ZnO NPs (100 mg/kg) (**C**), ZnO NPs (100 mg/kg) + esculetin (25 mg/kg) (**D**), ZnO NPs (100 mg/kg) + fucoidan (30 mg/kg) (**E**), esculetin (25 mg/kg) only (**F**), fucoidan (30 mg/kg) only (**G**). Scale bars = 200 μm.

Staining of the hippocampus of ZnO NP (10 and 100 mg/kg)-treated rats with anti-neuron-specific enolase (NSE) antibody showed the same histological architecture as the untreated controls (Figure 10A–C). In addition, there were no differences in labeling with anti-NSE antibody in the hippocampus between the combined ZnO NP and esculetin or fucoidan groups (Figure 10D,E), the groups treated with either esculetin or fucoidan alone (Figure 10F,G), and the untreated controls (Figure 10A).

### 3.10. Histological Examination of the Hippocampus with Anti-Iba-1 Antibody by ZnO NP Administration after Pre-Treatment of Esculetin or Fucoidan

**Figure 11:**
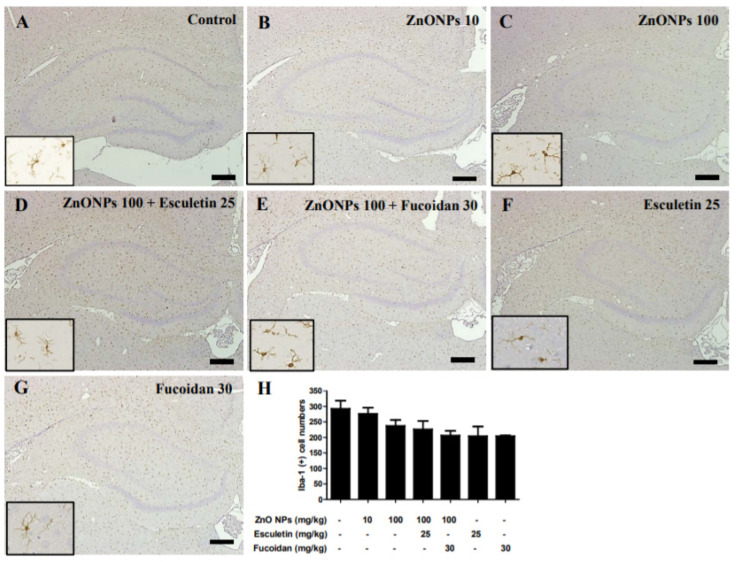
Immunostaining of ionized calcium binding adaptor molecule-1 (Iba-1) in the hippocampus of control and ZnO NP-treated rats with or without esculetin (25 mg/kg) or fucoidan (30 mg/kg) treatment. Control (**A**), ZnO NPs (10 mg/kg) (**B**), ZnO NPs (100 mg/kg) (**C**), ZnO NPs (100 mg/kg) + esculetin (25 mg/kg) (**D**), ZnO NPs (100 mg/kg) + fucoidan (30 mg/kg) (**E**), esculetin (25 mg/kg) only (**F**), fucoidan (30 mg/kg) only (**G**). Quantitative examination of Iba-1-positive cells in the hippocampus (**H**). Data represent the mean ± SEM (*n* = 3). All samples were counterstained with hematoxylin. Scale bars = 200 μm.

Immunoreactivity for Iba1, as a marker of microglial activity, was compared between ZnO NP-treated and untreated control rats to evaluate microglial activation in the hippocampus (Figure 11A–C). Immunoreactivity for Iba1 was detected in the hippocampus of all groups (Figure 11A–E). The number of Iba1-positive microglia in the hippocampus dose-dependently decreased in rats treated with ZnO NPs (276 ± 20 and 237 ± 19 in the 10 and 100 mg/kg ZnO NP groups, respectively) compared to untreated controls (292 ± 26) (Figure 11A–C). A semi-quantitative analysis showed that the numbers of Iba1-postive cells were slightly decreased in both 100 mg/kg ZnO NPs + 25 mg/kg esculetin (226 ± 26) and 100 mg/kg ZnO NPs + 30 mg/kg fucoidan (206 ± 15) groups compared to the 100 mg/kg ZnO NPs group (Figure 11C–E). The number of Iba1-postive cells decreased in the groups treated with esculetin (204 ± 31) and fucoidan alone (204 ± 2) compared to the untreated controls (Figure 11F,G). The insets in the left corner in each figure show higher magnification views from the same site of the hippocampus.

### 3.11. Immunohistological Examination of the Hippocampus with Anti-GFAP Antibody by ZnO NP Administration after Pre-Treatment of Esculetin or Fucoidan

**Figure 12:**
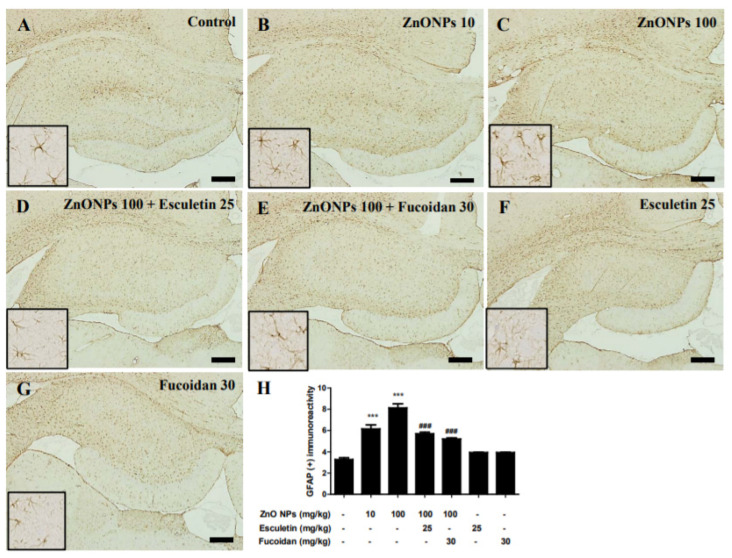
Immunostaining of glial fibrillary acidic protein (GFAP) in the hippocampus of control and ZnO NP-treated rats with or without esculetin (25 mg/kg) or fucoidan (30 mg/kg) treatment. Control (**A**), ZnO NPs (10 mg/kg) (**B**), ZnO NPs (100 mg/kg) (**C**), ZnO NPs (100 mg/kg) + esculetin (25 mg/kg) (**D**), ZnO NPs (100 mg/kg) + fucoidan (30 mg/kg) (**E**), esculetin (25 mg/kg) only (**F**), fucoidan (30 mg/kg) only (**G**). Semi-quantitative analysis of GFAP-positive area in the hippocampus (**H**). All samples were counterstained with hematoxylin. Data represent the mean ± SEM (*n* = 3). *** *p* < 0.001 compared to control, ### *p* < 0.001 compared to ZnO NPs (100 mg/kg) group. Scale bars = 200 μm.

**Figure 13:**
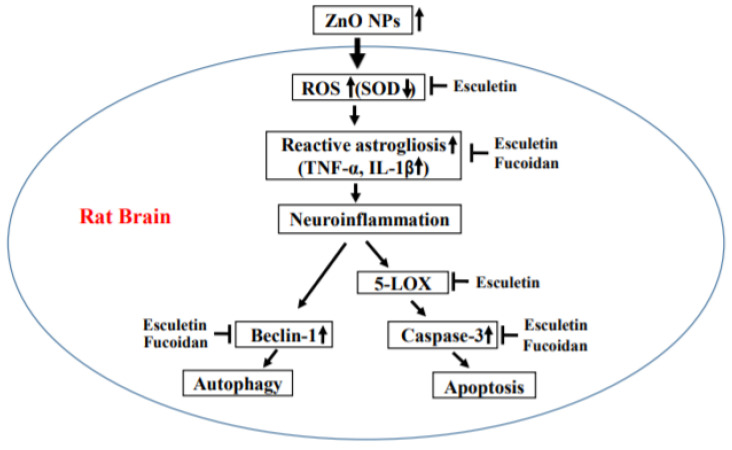
Possible mechanism of ZnO NP-induced neurotoxicity in the rat brain.

We measured the astrocyte activation with an evaluation of GFAP immunoreactivity. In the normal control group, GFAP immunoreactivity was detected in all regions of the hippocampus (Figure 12A). Treatment with ZnO NPs significantly increased GFAP immunoreactivity in a dose-dependent manner with GFAP-positive areas of 6.15 ± 0.69% in the 10 mg/kg ZnO NPs group and 8.12 ± 0.67% in the 100 mg/kg ZnO NPs group compared with the area of 3.27 ± 0.28% in the untreated controls (both *p* < 0.001) (Figure 12A–C). The increase in GFAP immunoreactivity by 100 mg/kg ZnO NP treatment was significantly ameliorated in both the 25 mg/kg esculetin and 30 mg/kg fucoidan group (5.71 ± 0.23% and 5.20 ± 0.20%, respectively, both *p* < 0.001) (Figure 12D,E). There have been reports of reactive gliosis induced by zinc or ZnO NPs during neuronal injury [22,26,54]. In this study, we demonstrated reactive astrogliosis in the rat hippocampus associated with oral ZnO NP administration, which was accompanied by swelling in the group treated with 100 mg/kg/day of ZnO NPs for 2 weeks. These dose-dependent changes were confirmed by a TEM analysis to occur in astrocytes, but not microglia, in the 10 and 100 mg/kg/day of ZnO NP treatment groups, without significant neurotoxic insults. These results were partially consistent with our previous reports indicating higher vulnerability of astrocyte than of neuronal cultures to injury by ZnO NPs [27,32]. Although microstructural analyses showed no substantial neurotoxicity—with TEM showing intact ER and mitochondria in neurons and NSE immunohistochemistry and H&E staining showing no abnormalities in the hippocampus—the elevation of autophagy and apoptosis by increased levels of proinflammatory cytokines, such as IL-6 and TNF-α, and the deficiency of the endogenous antioxidant enzyme, SOD, in brain homogenates were clearly observed. Astrocytes are the most abundant glial cells in the mammalian brain, and are known to play important roles in sustaining extracellular zinc levels in the CNS [55,56]. As reactive astrogliosis stimulates the release of pro- and anti-inflammatory cytokines, which in turn activate microglia and are related to the occurrence of secondary injuries [57,58], these results suggest that astrocytes may play more important roles in ZnO NP-induced brain injury than microglia. Further studies are required to determine the precise mechanism underlying the differences in activation between astrocytes and microglia by ZnO NPs. A possible hypothesis based on our results is illustrated in Figure 13.

Therefore, ZnO NP induced cerebral autophagy and apoptosis via reactive astrogliosis, oxidative stress, and proinflammatory cytokines without histological neurotoxicity. Esculetin and fucoidan are potential candidate compounds with which to improve cerebral injury by regulating the effects of ZnO NP on several neuropathological signs.

## 4. Conclusions

This study indicates that esculetin, an LOX inhibitor, and fucoidan, a marine algal polysaccharide, ameliorate ZnO NP-induced cerebral autophagy and apoptosis by regulating reactive astrogliosis accompanied by the swelling of astrocytes, but not microglia, as well as an increases in proinflammatory cytokines, IL-1β and TNF-α, and oxidative stress in the rat brain.

## Data Availability

The data presented in this study are available on request from the corresponding author.
